# A novel scale-space approach for multinormality testing and the *k*-sample problem in the high dimension low sample size scenario

**DOI:** 10.1371/journal.pone.0211044

**Published:** 2019-01-22

**Authors:** Kristian Hindberg, Jan Hannig, Fred Godtliebsen

**Affiliations:** 1 Department of Mathematics and Statistics, University of Tromsø – The Arctic University of Norway, Tromsø, Norway; 2 Department of Statistics and Operations Research, University of North Carolina, Chapel Hill, NC, United States of America; Universidad de Valladolid, SPAIN

## Abstract

Two classical multivariate statistical problems, testing of multivariate normality and the *k*-sample problem, are explored by a novel analysis on several resolutions simultaneously. The presented methods do not invert any estimated covariance matrix. Thereby, the methods work in the High Dimension Low Sample Size situation, i.e. when *n* ≤ *p*. The output, a significance map, is produced by doing a one-dimensional test for all possible resolution/position pairs. The significance map shows for which resolution/position pairs the null hypothesis is rejected. For the testing of multinormality, the Anderson-Darling test is utilized to detect potential departures from multinormality at different combinations of resolutions and positions. In the *k*-sample case, it is tested whether *k* data sets can be said to originate from the same unspecified discrete or continuous multivariate distribution. This is done by testing the *k* vectors corresponding to the same resolution/position pair of the *k* different data sets through the *k*-sample Anderson-Darling test. Successful demonstrations of the new methodology on artificial and real data sets are presented, and a feature selection scheme is demonstrated.

## Introduction

In practice, it is frequently assumed that a data set can be described by a multivariate normal distribution. Many common statistical procedures rely on the data being multinormal, something which is often not adequately checked before using the procedures [1–3]. Often, this assumption is false for either the whole data set or parts of it. Another classical problem is the testing of whether *k* multivariate data sets originate from the same distribution. For each of the two problems, a scale-space inspired algorithm that tests all resolutions and positions simultaneously, is presented. See the “Materials and methods” section for definitions of “resolution” and “position”. The two presented algorithms are very similar apart from the type of one-dimensional tests used. A weighted summation is performed across the dimensions/positions in both algorithms. The notion of resolution is connected to the number of dimensions being summed across, while the different dimensions/positions typically are temporal or spatial samples.

**Fig 1:**
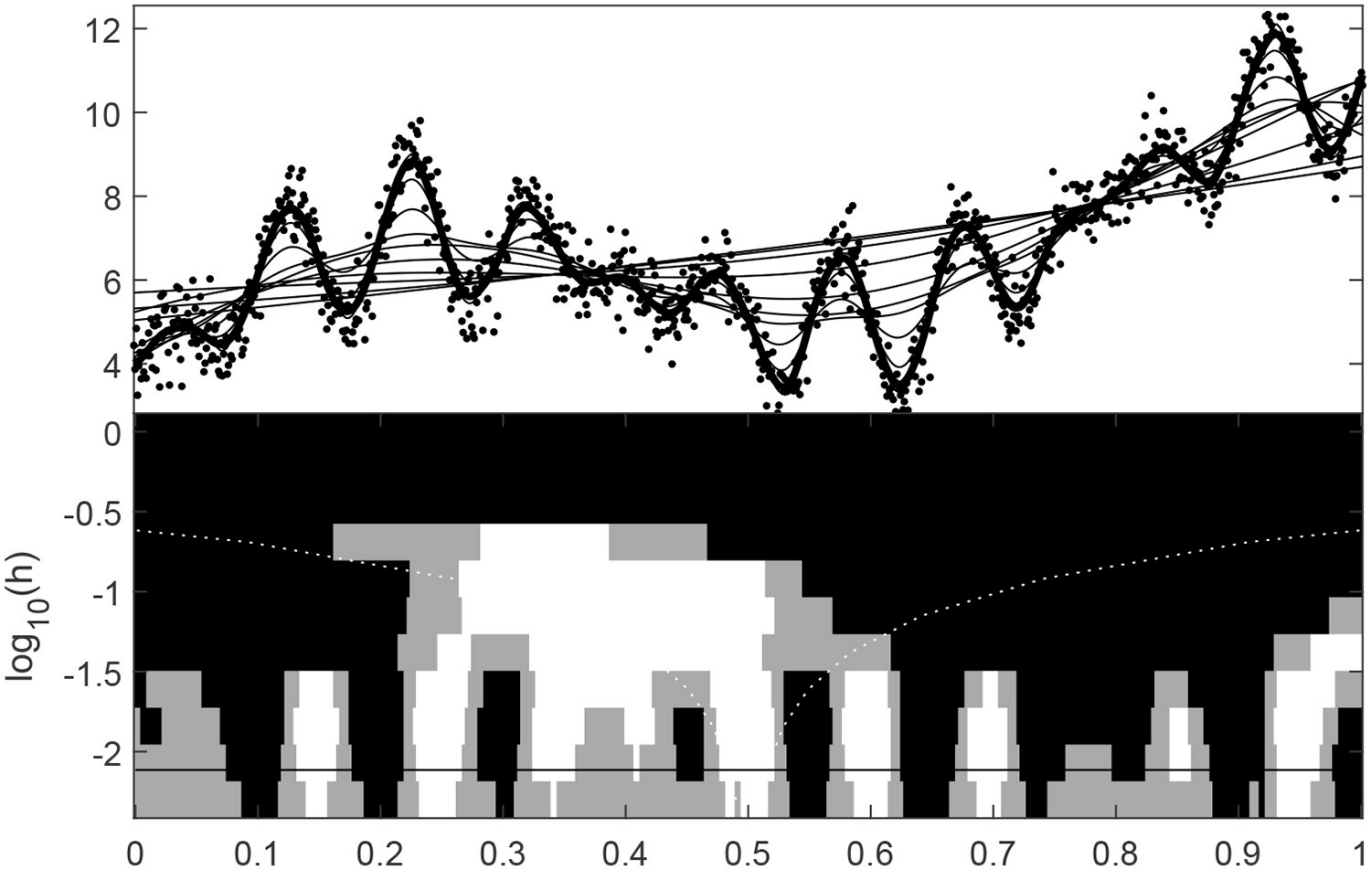
SiZer map of artifical data. The upper panel shows the artifical values as dots and a set of smoothed curves with different smoothing bandwidths. The solid line, which is very close to the true underlying signal, corresponds to a computer-chosen optimal bandwidth. In the lower panel, the vertical axis corresponds to, from top to bottom, wider to narrower smoothing bandwidths (the horizontal line corresponds to the computer-chosen optimal bandwidth). White and black pixels correspond to significant decrease and increase, respectively. Gray pixels correspond to situations where the background signal can be assumed constant.

Scale-space theory is a framework for representing signals on multiple scales/resolutions, developed by the computer vision, image processing and signal processing communities. The development of scale-space methodology is typically regarded to start with two papers by Witkin [4, 5]. A recent review by Holmström and Pasanen [6] shows how scale-space methodology has been extended to a large number of areas. The goal of statistical scale-space methodology is to extract features from noisy data at several levels of resolution. Typically, the data is an observed time series or a digital image where features at different temporal or spatial scales/resolutions might be of interest. Since the scale-space idea is important in the present paper, we introduce the scale-space idea through the SiZer methodology developed by Chaudhuri and Marron [7]. To this end, we produce the output from SiZer in Fig 1 when applied to an artificial data set. SiZer is based on nonparametric smoothing and the upper panel shows the artificial data points as dots and a large number of curves obtained for different values of the smoothing parameter. In this setting, the scale/resolution corresponds to the value of the applied smoothing parameter. A rough curve in the upper panel corresponds to a small smoothing parameter and hence to a short scale. Long scales correspond to smooth curves obtained by large values of the smoothing parameter. The SiZer map in the lower panel reveals what features the observed data contain at different scales. In this context, a black pixel means that the curve is significantly increasing, a white pixel corresponds to a significantly decreasing feature, and a gray pixel corresponds to a situation where the curve is considered to be flat. From Fig 1, it can be seen that SiZer flags regions as significantly decreasing and/or increasing for different positions and smoothing parameters.

In the present paper, we will adapt the SiZer methodology to our situation and develop a scale-space methodology that can be useful for the *k*-sample problem and for testing of multinormality.

The presented algorithms have two aspects that make them useful in many situations. As will be shown, the algorithms avoid the need to estimate the covariance matrix, leading to algorithms that can handle the High Dimension Low Sample Size (HDLSS) situation. Furthermore, the algorithms allow an evaluation of the data set for all resolutions and all positions simultaneously. By this approach, it may, for the multinormality testing, be detected if only some parts of the data set originate from a multinormal distribution. For the *k*-sample case, the scale-space approach can detect if one or more of the *k* samples differ on different resolutions and/or positions. By not estimating the covariance matrix, the presented scale-space tests potentially loose some power compared to tests that incorporate the information from the estimated covariance matrix. This loss of power is acceptable on the grounds of being able to handle the HDLSS situation. As a result of the summation, the algorithms will include a large number of one-dimensional tests. Two versions of the Anderson-Darling (AD) test (see the “Anderson-Darling testing” section) are applied as one-dimensional tests for the multinormality testing problem and the *k*-sample problem. The choice of using the AD test is a result of its excellent power against all alternatives and existence of very good approximations for the asymptotic distribution and formulas adjusting for the finite sample sizes [8–10].

For the results presented, the Anderson-Darling (AD) test (see the “Anderson-Darling testing” section) is used for both the multinormality testing and the *k*-sample problem as the one-dimensional test used on the summations. The choice of using the AD test is a result of its excellent power against all alternatives and existence of very good approximations for the asymptotic distribution and formulas adjusting for the finite sample sizes [8–10].

**Fig 2:**
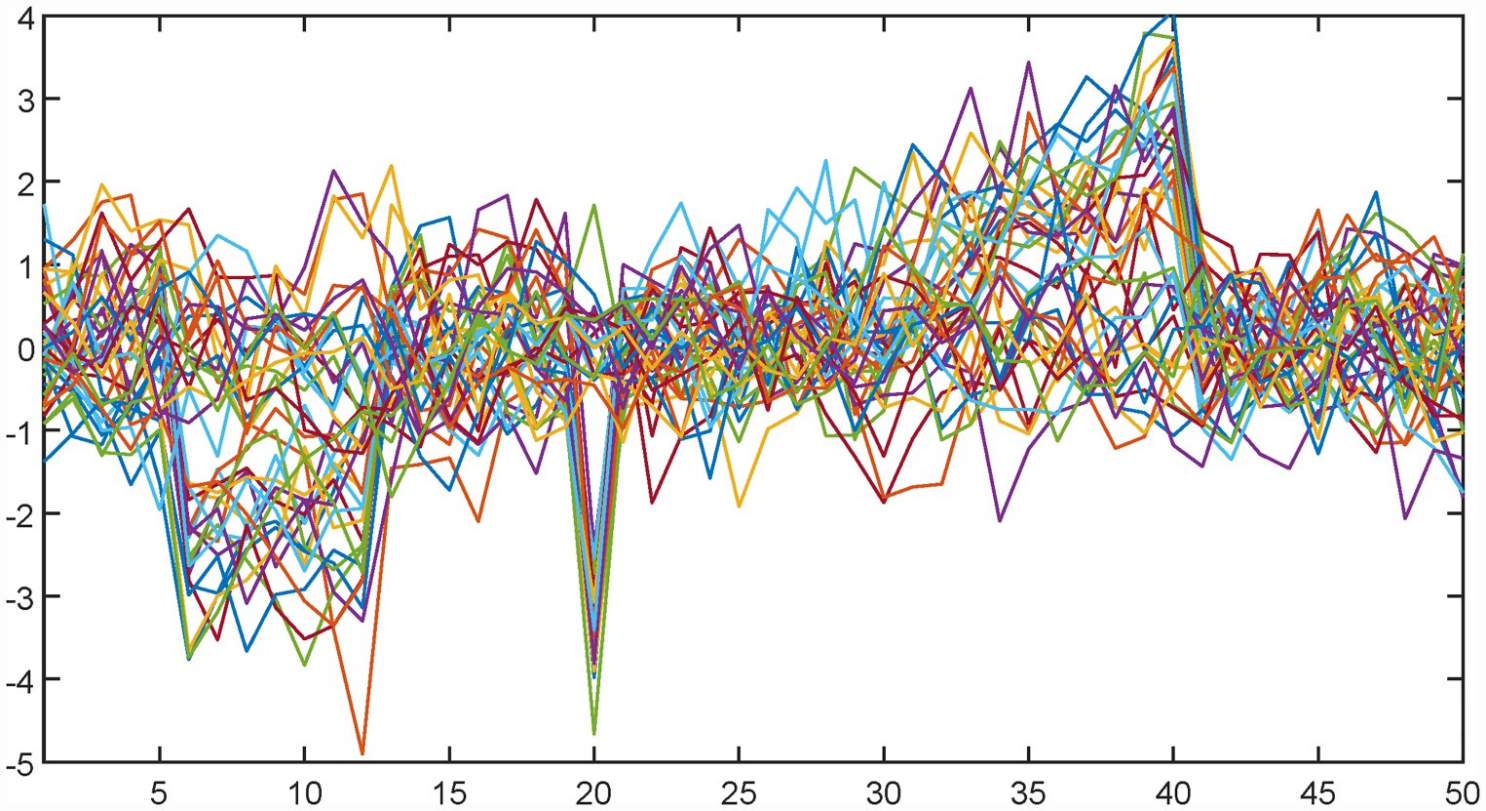
All 40 artificial signals of length 50.

A simple artificial example is presented to illustrate the main ideas of the paper. The data set is generated to have a distribution that is multivariate normal for some of the dimensions and a mixture of two different normal distributions for the rest of the dimensions. In particular, the population is a mixture of two different underlying true signals. In the first population, 20 signals are sampled from a zero mean Gaussian autoregressive process of order 1, more specifically cov(*X*_*i*_, *X*_*j*_) = 0.5^1+|*i*−*j*|^. The remaining 20 signals have the same covariance structure, but a different mean. In particular, the mean of the second population is equal to −2.15 for position 6 to 12 and −3.5 for position 20. For indices 26, …, 40, the expected value increases linearly from 0.1 to 2.5, while the rest of the dimensions have expectation equal to zero. Fig 2 shows all the 40 signals of length 50.

**Fig 3:**
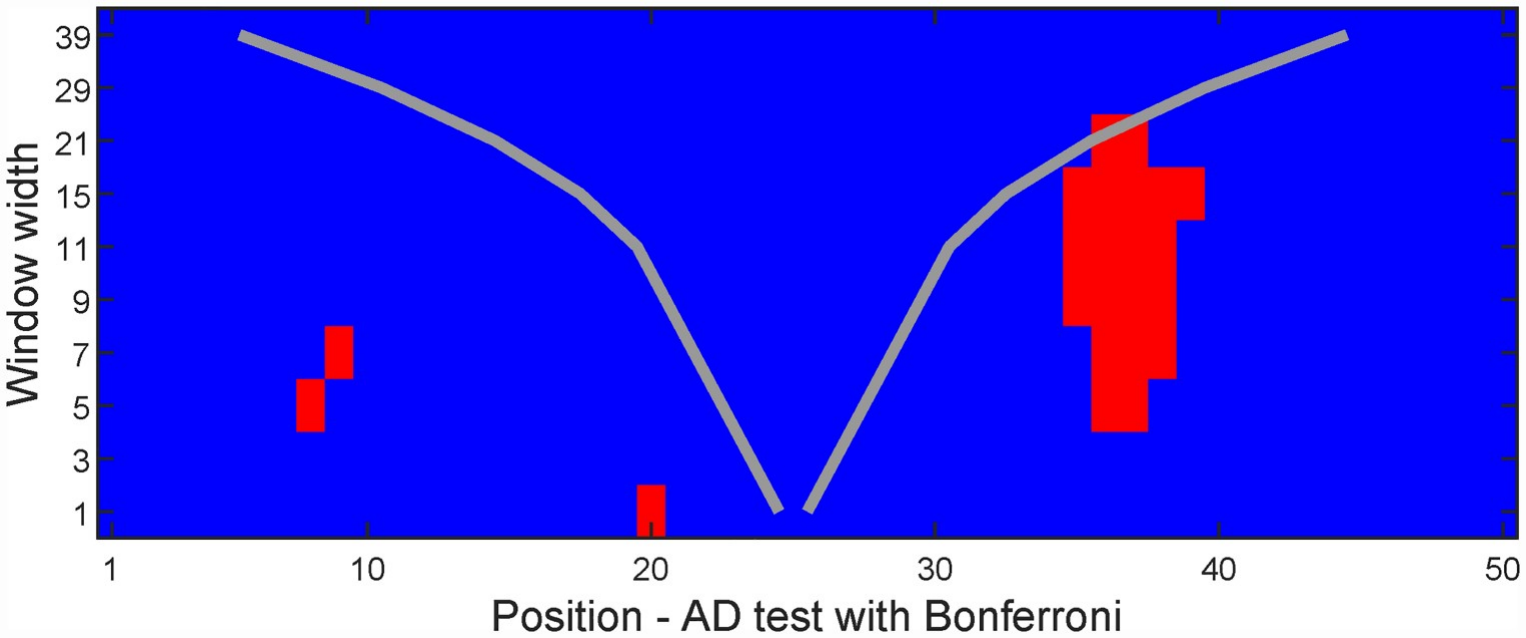
Significance map of the test for multinormality of an artificial data set. Red indicates rejection of the null hypothesis (multinormality) for that window width/position. For a given resolution, the horizontal distance between the two gray lines equals the width of the summation window of that resolution.

Fig 3 shows the resulting significance map from the proposed multinormality test of the data in Fig 2. The horizontal axis is the same as in Fig 2 and shows the position, while different window widths are given on the vertical axis. These different window widths represent the resolution part of the presented algorithms. Resolution 1 corresponds to testing the marginal distribution of each dimension. Higher resolutions are results of normality tests of local averages at a corresponding position and corresponding window width. For a distribution to be multinormal, all marginals and all local averages must be normally distributed. By going through the test results for all resolution/position pairs, the significance map is produced. Red pixels mark Bonferroni [11] adjusted rejections of the null hypothesis of normality, i.e. indicating that the part of the data matrix that is summed across cannot be considered as a sample from a multinormal distribution. Note that the abrupt deviation from normality at dimension 20 is found on low resolution values, while the more gradual departure from multinormality at dimension 6 to 12 and dimensions 25 to 40 are found on larger resolution values. This example shows that both low and high resolutions may be of importance in the same data set.

The “Materials and methods” section presents the concept of scale/resolution and space/position as used in this paper, the statistical problems being investigated and the details of the two presented algorithms. Some investigations into the power of the tests are also presented. In the “Results” section, the algorithms are applied to some real data sets, comparisons with other algorithms are done, and a feature selection scheme is presented and tested on real data. Finally, the “Conclusions” section sums up the presented methods.

## Materials and methods

Recall that an important motivation for applying a scale-space approach is the fact that different phenomena can be visible/detectable on different resolutions and/or positions of the data set. In classical nonparametric smoothing schemes, some sort of bandwidth parameter has to be chosen [12]. By selecting one bandwidth only, features detectable on other bandwidths will not be found. However, using a scale-space approach, one can look at all bandwidths simultaneously. Scale-space ideas have proven useful in many areas and have been applied to feature detection in curves and images [7, 13], density estimation [14], curve fitting [15], Bayesian time series analysis [16] and spectral feature detection [17].

### Assumptions

For the multinormality testing case, let **X**_1_, **X**_2_, …, **X**_*n*_ be a set of *p*-dimensional vectors. The null hypothesis assumes that these vectors originate from a *p*-dimensional multinormal distribution $N(\mu ,\underline{\Sigma })$, i.e.

$$\begin{array}{c} H_{0}\;:\;X_{i}∼N\left(\mu ,\underline{\Sigma }\right)\;\forall \;i, \end{array}$$

where the mean vector ***μ*** and the covariance matrix $\underline{\Sigma }$ are unknown. For the presented algorithm, the parameters of this assumed multinormal distribution do not have to be estimated. Note that by avoiding the need for an estimate of the covariance matrix, the algorithm can be applied to data sets with any combination of sample size and sample dimension, as long as the sample size is high enough for the one-dimensional normality test to be applicable.

The algorithm works with any covariance structure and there are no requirements for smoothness of expected values of neighboring dimensions. As will be presented later, the algorithm performs a weighted summation across neighboring dimensions. A motivation behind this summation is that neighboring dimensions frequently have some sort of logical connection to each other, as for example in a time series. When the data set consists of a time series, the different dimensions are equivalent to the different sampling times. If the dimensions are shifted around, the algorithm could produce different results. Therefore, interpretations of the results are easier when the different dimensions have a natural ordering, as for example with spatial or temporal data.

For the *k*-sample case, each of the *k* samples consist of a given number (which can be different for each *k*) of *p*-dimensional vectors with unknown cumulative distribution functions (CDF), given by *F*_1_, *F*_2_, …, *F*_*k*_, respectively. The null hypothesis is then stated as

$$\begin{array}{c} H_{0}\;:\;F_{1}\left(x\right)=F_{2}\left(x\right)=\cdots =F_{k}\left(x\right),\;\forall \;x\in R^{p}. \end{array}$$ (1)

Since this methodology only tests whether or not the CDFs all are the same, the CDFs can take any form or belong to any class of distributions. Again, the interpretations of the results are easiest when working with data having a natural ordering.

### Concept of resolution and summation across dimensions

One of the main ideas of this manuscript is testing simultaneously for many different resolutions and positions. The resolution value equals the number of different dimensions being summed across. The lowest resolution value of 1 corresponds to a test of the marginal distributions. At resolution 3, the result of the summation for position/dimension *d* is a weighted (see Eq (2)) summation of the sample values with position index *d* − 1, *d* and *d* + 1. For other resolutions, completely analogous summations are performed. Note that by this summation, small differences within the data can be detected, even though this difference might not be detected for lower resolutions. The set of default resolutions is chosen to be {1 3 5 7 9 11 15 21 29 39 51 65 81 99 … *s*_max_}, i.e. for *i* ≥ 5 the resolution values are given as *s*_*i*+1_ = *s*_*i*_ + 2 ⋅ (*i* − 4) up to a maximum resolution *s*_max_ ≤ *p*, where *s*_5_ = 9. Alternatively, one can choose to only include resolutions up to some upper resolution.

**Fig 4:**
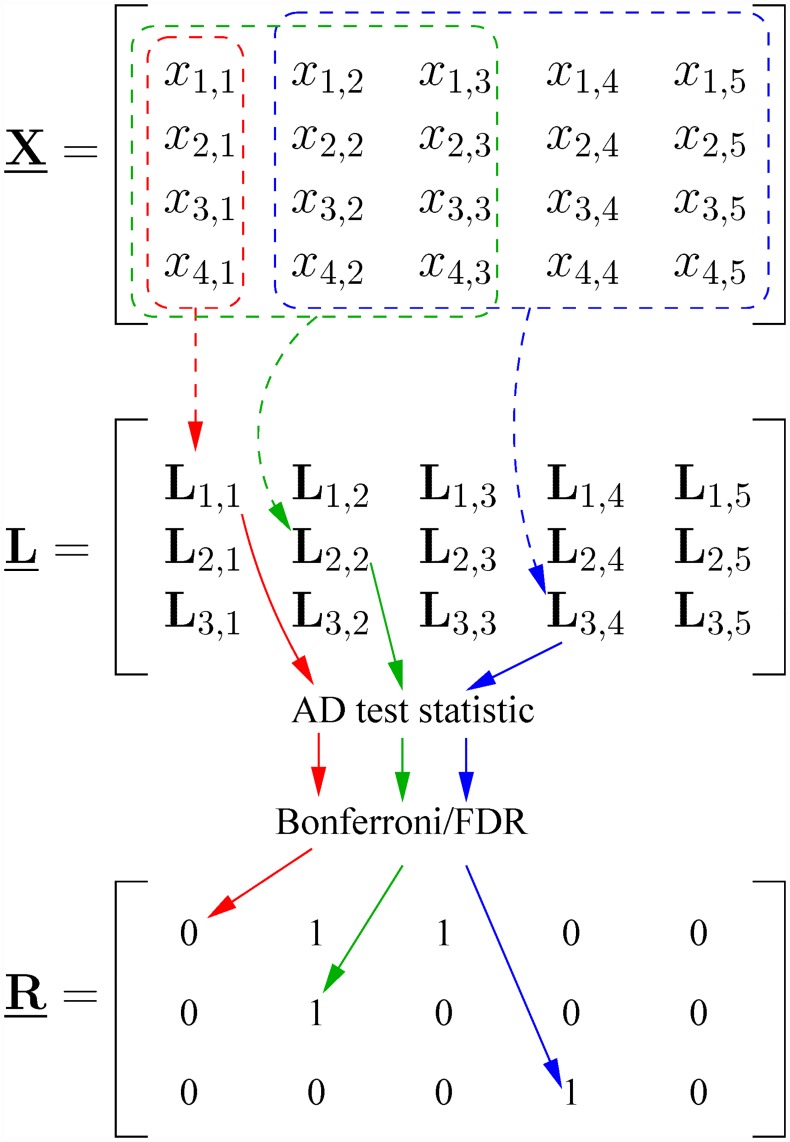
Workflow chart. The data matrix $\underline{X}$ has dimensions [*n*, *p*] = [4, 5]. The summation matrix $\underline{L}$ has dimensions [*n*_*s*_, *p*, *n*] and each **L**_*s*,*d*_ is a vector of length *n*. The significance matrix $\underline{R}$ has dimensions [*n*_*s*_, *p*]. The red box, which only spans one dimension, indicates that for the lowest resolution value, no summation is performed across the dimensions. For the green and blue boxes, summation is performed across dimensions 1–3 and 2–5, respectively. The blue box is adjusted to not extend outside the data matrix. Note that two significance maps are produced, one each for the Bonferroni/FDR approaches, with ones in $\underline{R}$ marking rejections of the null hypothesis for the corresponding resolutions and positions. When plotting the significance maps, the vertical axis is inverted.

For each of the different resolutions *s*, a weighted summation across different dimensions/positions is performed, producing **L**_*s*,*d*_, where *d* is the position index ranging from 1 to *p* and **L**_*s*,*d*_ is a vector of length *n*. The resulting **L**_*s*,*d*_’s form a matrix $\underline{L}$ with size [*n*_*s*_, *p*, *n*], where *n*_*s*_ is the number of resolutions being used. A discrete Epanechnikov [12] window function is used as summation weights. For a given pair of *s* and *d*, the Epanechnikov summation window is a column vector given by

$$\begin{array}{c} w_{s,d}\left(i\right)\equiv K\cdot {\left[1-{\left(\frac{i-d}{\left\lceil s/2\right\rceil }\right)}^{2}\right]}_{+},\;\;\;\;\;i=1,\ldots ,p, \end{array}$$ (2)

where *K* is some normalizing constant, ⌈⋅⌉ is the ceiling function, and the plus function is defined as [*f*(*x*)]_+_ ≡ max[0, *f*(*x*)] for some functional value *f*(*x*). The **L**_*s*,*d*_ vector is generated through

$$\begin{array}{c} L_{s,d}=\underline{X}\cdot w_{s,d}, \end{array}$$

where the data matrix $\underline{X}$ has size [*n*, *p*], with the *n* samples of length *p* along each row, and ⋅ indicates normal matrix multiplication. The resulting vector **L**_*s*,*d*_ is thereby a weighted summation across the *s* dimensions centered on the *d*-th dimension. Fig 4 shows how the algorithm generates the $\underline{L}$ matrix and how it is used to generate the output matrix, that is the significance map $\underline{R}$, with different resolutions on the vertical axis and position on the horizontal axis.

As an example one can calculate the vector elements of the $\underline{L}$ matrix corresponding to the resolution/position pairs (1, 1), (2, 2) and (3, 4) of the data matrix

$$\begin{array}{c} \underline{X}=[\begin{array}{ccccc} 0 & 0 & 0 & 1 & 1\\ 0 & 1 & 1 & 3 & 2\\ 1 & 1 & 0 & 1 & 1\\ 2 & 1 & 1 & 0 & 0 \end{array}]. \end{array}$$

The Epanechnikov weights for the given resolution/position pairs equal **w**_1,1_ = [1, 0, 0, 0, 0]^*T*^, **w**_2,2_ = 1/10 ⋅ [3, 4, 3, 0, 0]^*T*^, and **w**_3,4_ = 1/30 ⋅ [0, 5, 8, 9, 8]^*T*^, where ^*T*^ indicates the transpose. The resulting vector elements are

$$\begin{array}{c} L_{1,1}={\left[0,0,1,2\right]}^{T}\;\;,\;\;L_{2,2}=\frac{1}{10}\cdot {\left[0,7,7,13\right]}^{T}\;\;,\;\;L_{3,4}=\frac{1}{30}\cdot {\left[17,56,22,13\right]}^{T}. \end{array}$$

### Normality testing

From the matrix $\underline{L}$, the actual one-dimensional normality test statistics are calculated. For each of the (*s*, *d*) pairs, the p-value of the AD test statistic of the vector **L**_*s*,*d*_ is stored. To address the problem of multiple testing, the algorithm outputs two significance maps, one based on the Bonferroni approach and one based on False Discovery Rate (FDR) [18]. The *p*-dimensional vector of p-values of each resolution is fed into FDR, generating the FDR-based significance map resolution by resolution. For the Bonferroni approach, the critical value is obtained from the nominal significance level *α* divided by the number of dimensions *p*, producing on average one false alarm every 1/*α* resolution. This follows the usual SiZer recommendation of adjusting the significance for each resolution separately. The alternative, adjusting the output map for all the resolution/position pairs simultaneously, is known from the SiZer literature to be overly conservative [7]. The nominal significance level is by default equal to *α* = 0.05.

### The *k*-sample problem

For the *k*-sample problem, the *k* data matrices ${\underline{X}}_{i},\;i=1,\ldots ,k$ are all put through the summation procedure of Fig 4, producing ${\underline{L}}_{i},\;i=1,\ldots ,k$. For each resolution/position pair (*s*, *d*), the *k* corresponding vectors (of size *n*_*i*_, *i* = 1, …, *k*) from the ${\underline{L}}_{i}$ matrices are fed into the *k*-sample AD test [9, 19]. The distributions of the sums along the dimensions will in general be different from the marginal distributions. Nevertheless, if the *k* data sets do have the same multivariate distribution, for a given resolution/position pair (*s*, *d*), the distributions of the *k* different summation vectors will be the same. The p-values of the tests are stored and used in the generation of the FDR-based significance map, while the Bonferroni approach finds the critical value as for the multinormality testing. If the null hypothesis is rejected, the (*s*, *d*)-element of the output matrix is marked as a significant element, indicating that at least one of the empirical distributions are significantly different from the others for this resolution/position pair.

### Anderson-Darling testing

The two algorithms presented use three different AD tests. The AD goodness-of-fit test is used in the case of checking for multinormality [20–22]. For the two-sample/*k*-sample case, the versions of the AD test suggested by [9] and [19], respectively, are used.

The AD goodness-of-fit test checks the simple null hypothesis that a sample is from a distribution with a known continuous CDF, *F*(*x*). Let *x*_1_ ≤ *x*_2_ ≤ ⋯ ≤ *x*_*n*_ be the ordered sample of size *n*, and let *u*_*i*_ = *F*(*x*_*i*_), *i* = 1, …, *n*. The AD test statistic is defined as

$$\begin{array}{c} A_{n}^{2}\equiv -n-\frac{1}{n}\sum_{i=1}^{n}\left(2i-1\right)\ln[u_{i}\left(1-u_{n-i+1}\right)]. \end{array}$$ (3)

This clearly shows that the AD test is distribution free, as long as the null distribution is fully known. Approximate expressions for the asymptotic distribution of the AD test are given by [8, 10].

When testing for multinormality with unknown distributional parameters, i.e. testing a composite hypothesis, *F*(*x*) is some unknown normal CDF, something which changes the distribution of the AD test statistic. In this case, the sorted data are normalized, producing *z*_*i*_, *i* = 1, …, *n*. Then, $u_{i}^{\prime }=F_{0}\left(z_{i}\right)$ is produced, where *F*_0_(⋅) is the standard normal CDF. These $u_{i}^{\prime }$ values are fed into Eq (3), and the final test statistic is obtained by applying the correction factor for finite sample sizes given on page 123 of [23]. The p-values and critical values are calculated from the approximations given on page 127 of [23]. Following page 373 of [23], the presented algorithm requires *n* ≥ 8. The presence of ties in the data is a good indicator of non-normality, something which the AD test will reflect too. For instance, if normally distributed data is in some way rounded off, the rejection rate will be higher than the rate expected from the prescribed significance level.

For the *k*-sample case, there is no need to estimate any parameters, and the test statistic reduces to a rank statistic. Hence, under the null hypothesis, the distribution of the test statistic is independent of the distribution of the *k* samples. The two-sample case and the *k*-sample case are treated separately, even though the *k*-sample reduces to the two-sample case in [9] when *k* = 2. The correction factor in [9] is used to produce the final two-sample test statistic. [9] shows that the distribution of the sample-size adjusted two-sample AD test statistic can be approximated well by the asymptotic distribution of the AD goodness-of-fit test for a fully known null distribution. The presented algorithm uses Equation (3.6) in [10] to produce the approximate p-value of the test statistic when *k* = 2.

The general *k*-sample AD test statistic in [19] is given as

$$\begin{array}{c} A_{kN}\equiv \frac{1}{N}\sum_{i=1}^{k}\frac{1}{n_{i}}\sum_{j=1}^{N-1}\frac{{\left(NM_{ij}-jn_{i}\right)}^{2}}{j(N-j)}, \end{array}$$

where *N* = *n*_1_ + *n*_2_ + ⋯ + *n*_*k*_, and *M*_*ij*_ is the number of observations in the *i*-th sample that are not greater than the *j*-th observation of the pooled sample of all *k* samples. Equation (6) in [19] modifies the expression for *A*_*kN*_, to be able to handle ties in the data. The presented algorithm uses the expression adjusted for ties, both for the two-sample and *k*-sample cases. Thereby, *F*_*i*_(*x*) in Eq (1) can be connected to a continuous or discrete random vector. The interpolation scheme of [19] is used to determine the p-value of *A*_*kN*_ when *k* > 2. Inspired by [9], it is required that all *n*_*i*_ ≥ 8, *i* = 1, …, *k*.

In theory, any omnibus, univariate test that achieves a specified significance level can be used in the presented framework for testing the results of the weighted summations. Relying on power studies by [24–26], the well-known, univariate Shapiro-Wilk test [24, 27, 28] is seen as the best alternative to the univariate AD test used for multinormality testing. Other tests that were considered include Watson’s *U*^2^ test [29], Kuiper’s test [30], Lilliefors’ test [31], the Cramér-von-Mises test [32], the Shapiro-Francia test [33], D’Agostino-Pearson’s *K*^2^ test [34, 35], the Jarque-Bera test [36], and Doornik’s test [37]. Other tests considered for the *k*-sample case include the Kolmogorov-Smirnov test [38], the Cramér-von-Mises test [38], and Watson’s $U_{k}^{2}$ test [39].

### Cramér-Wold

The Cramér-Wold theorem states that two random column vectors **X** and **Y** have the same distribution if and only if for all row vectors **a**, the random variables **a** ⋅ **X** and **a** ⋅ **Y** have the same distribution [40]. In the presented algorithms, the different summation weights of the Epanechnikov window take the role of **a**. Thereby, when doing the summation and testing for normality/difference between samples for many resolutions, a set of **a** vectors are applied to the single or many data sets. The Cramér-Wold theorem requires that the distribution of **a** ⋅ **X** and **a** ⋅ **Y** are equal for all possible **a** vectors. In the presented setting, only a finite number of vectors are tested. Since the presented algorithms are most suitable for data with some sort of neighboring structure (e.g. time series or spatial data), the important **a** vectors should be those that look at dimensions close to each other to a varying degree. Hence, following the Cramér-Wold theorem, a lack of rejection for (almost) all resolutions/positions should be seen as a good indication of the null hypothesis actually being true for the whole data set.

### Significance of rejections

**Fig 5:**
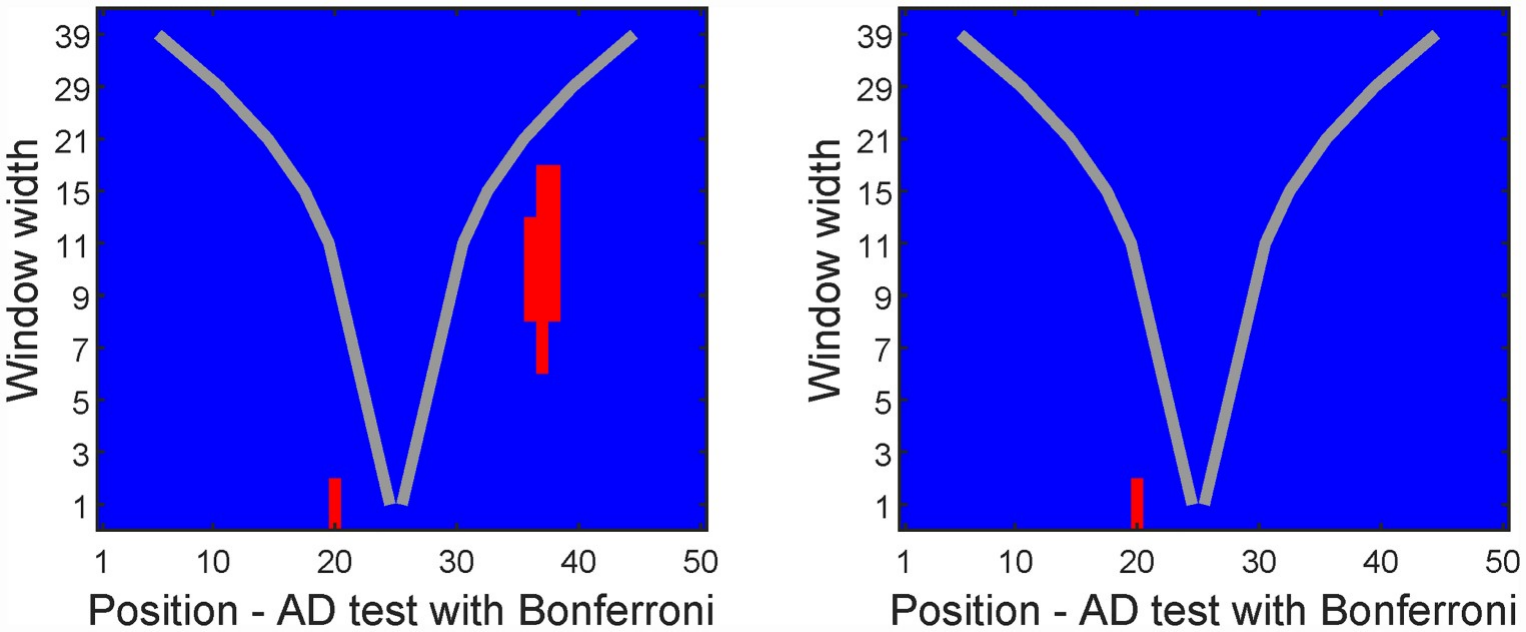
Significance maps of the scale-space multinormality test for the data of the Introduction. Left/Right: Significance level of 0.005/0.001.

The p-value is available for all the resolution/position pairs. The lower the p-value of a “rejection pair”, the more significant the rejection of the null hypothesis is on that resolution/position. By changing the significance level, one can determine on which resolution/position the null hypothesis is most significantly rejected. In Fig 5 the example of the Introduction is revisited, where significance levels of 0.005 and 0.001 are used, compared to 0.05 in the Introduction. By comparing Fig 5 to Fig 3, it is clear that for this realization, the most significant region is the single non-normal dimension of position index 20, and the region from index 26 to 40 is the second most non-normal.

### Power of the scale-space tests

**Fig 6:**
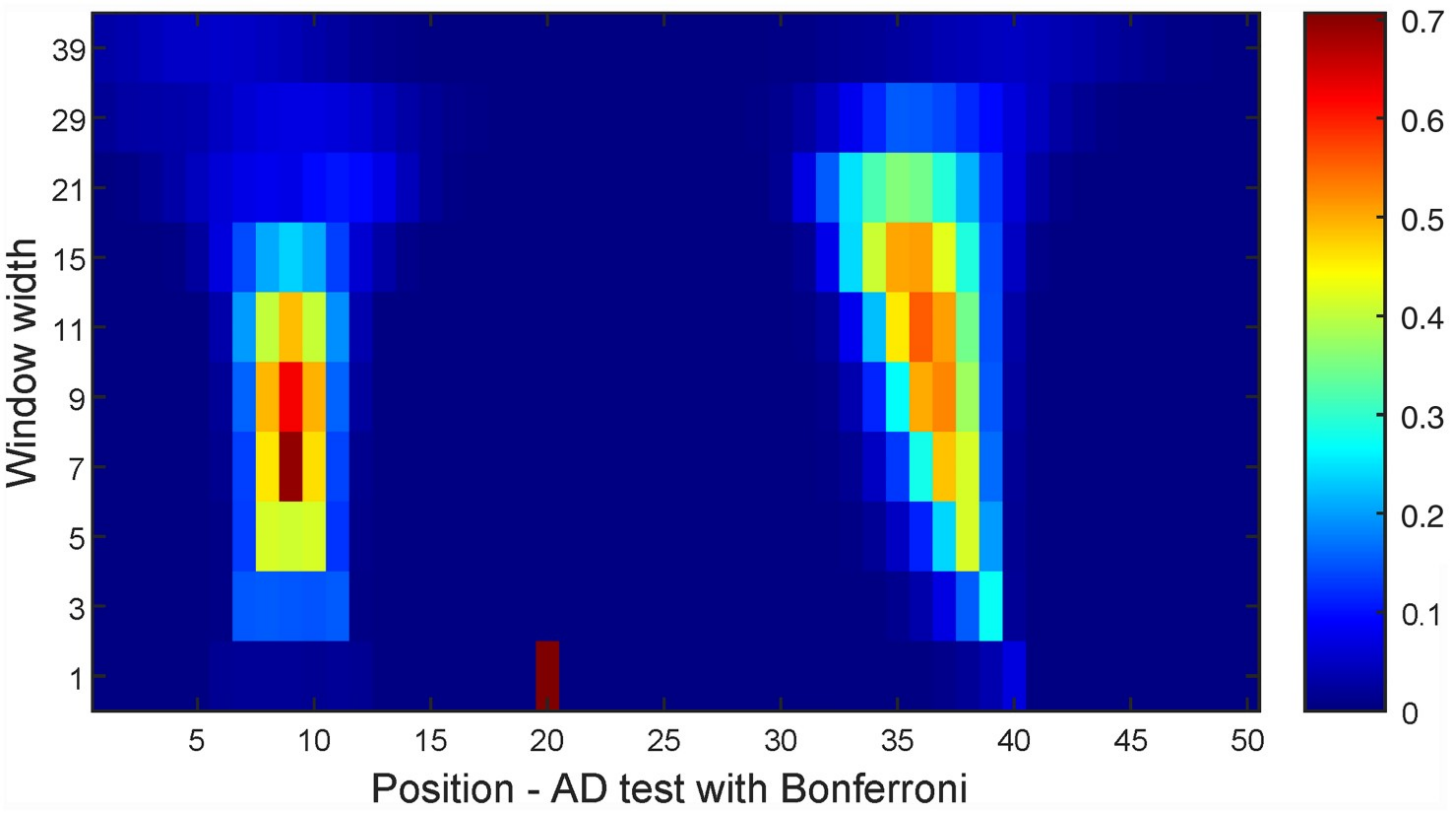
Rejection ratios of all resolution/position pairs for 1 000 replications of the motivational example.

There are no clear templates for power studies of the proposed scale-space tests. After the summations are done, the tests use the well-documented AD tests. Thereby, the power of the scale-space tests is connected to the power of the AD tests. Instead, it can be informative to illustrate how the power varies over the different resolution/position pairs of the output matrix for a given example. Assume that the data set has the same structure as in the motivational example of the Introduction. Fig 6 shows the rejection ratio (from 1000 data sets) of the scale-space test for multinormality. As can be seen, one finds the highest powers for the resolution/position pairs that best fit the non-normal dimensions. Similar results would be obtained for the test for comparing *k* data sets.

To investigate the effect of increased number of dimensions, a number of normally distributed dimensions are added to the right side of the signal of the Introduction. Table 1 shows the power of the multinormality test for different number of dimensions and for the FDR/Bonferroni correction. The case of 50 dimensions in total corresponds to the power of the pairs of Fig 6. From this it is clear that the power decreases as the number of dimensions grows, which is to be expected as adjustments for multiple testing are enforced.

**Table 1:** Power of test for multinormality when the signal of the Introduction is augmented with a number of normally distributed dimensions.

	Window width/position pair					
	1/20		7/9		9/37	
Dimensions in total	FDR	Bonf.	FDR	Bonf.	FDR	Bonf.
50	0.735	0.687	0.863	0.684	0.808	0.563
100	0.619	0.583	0.773	0.596	0.668	0.440
250	0.457	0.443	0.578	0.433	0.481	0.272
500	0.331	0.312	0.411	0.295	0.334	0.197
1000	0.264	0.248	0.298	0.219	0.227	0.116

## Results

The presented algorithms are tested on a number of different data sets. A five percent significance level is used for all the figures, unless otherwise stated. First, the initial example of the Introduction is investigated in more detail.

### Introductory example revisited

For larger resolutions, the scale-space test for multinormality can be shown to increase the mode separation if the distribution has more than one mode. This is demonstrated through some simple examples. Assume that all the dimensions of some data set are unimodal normal with different means and/or variances for different dimensions. The result of the summation will then be some other normal distribution.

A short example of this is given. Assume that the data matrix $\underline{X}$ has dimensions [10, 3] and that column 1, 2, and 3 contain N(0,1), N(4,1), and N(8,1), distributed variables, respectively. The summation (for simplicity, assuming even weights of 1/3) over these three columns would produce a 10-element long vector with distribution N(4,1/3), which the AD test would detect as normal, i.e. the test would not reject it.

Now assume that the ten samples of a given dimension do not have the same distribution. Assume that the five first samples of the three columns are distributed as N(1,1), while the last five are distributed as N(0,1). When checking the columns separately, the 10-element vector might not “look” enough different from a unimodal normal distribution to be rejected by the AD test. When summing (again, assuming even weights of 1/3) over the three columns, the distribution of the sum of the first five samples is given by N(1,1/3), while the last five have a N(0,1/3) distribution. This shows that the peaks have larger separation (both variances have decreased) as a result of the summation.

### Multinormality of temperature data

**Fig 7:**
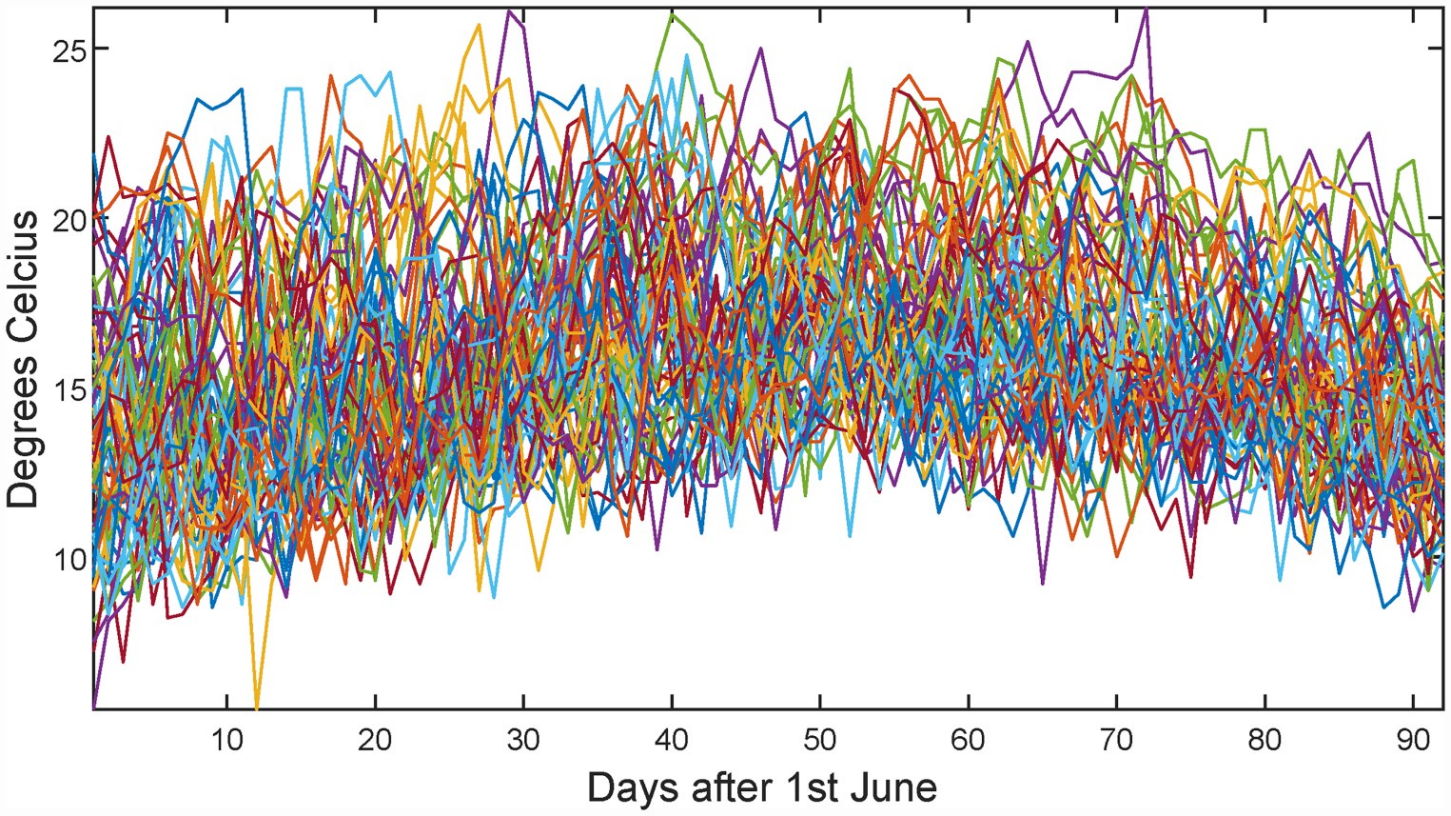
Daily mean temperatures at Oslo, Blindern, for the period 1937-2008.

A data set obtained from the Norwegian Meteorological Institute is analyzed. The data show daily mean temperature for the 92 days of June–August for the period 1937 to 2008 at Blindern, Oslo. This gives a data matrix of dimensions [*n*, *p*] = [72, 92], making algorithms that rely on inversion of the estimated covariance matrix impossible to use. A plot of all the 72 years is given in Fig 7.

**Fig 8:**
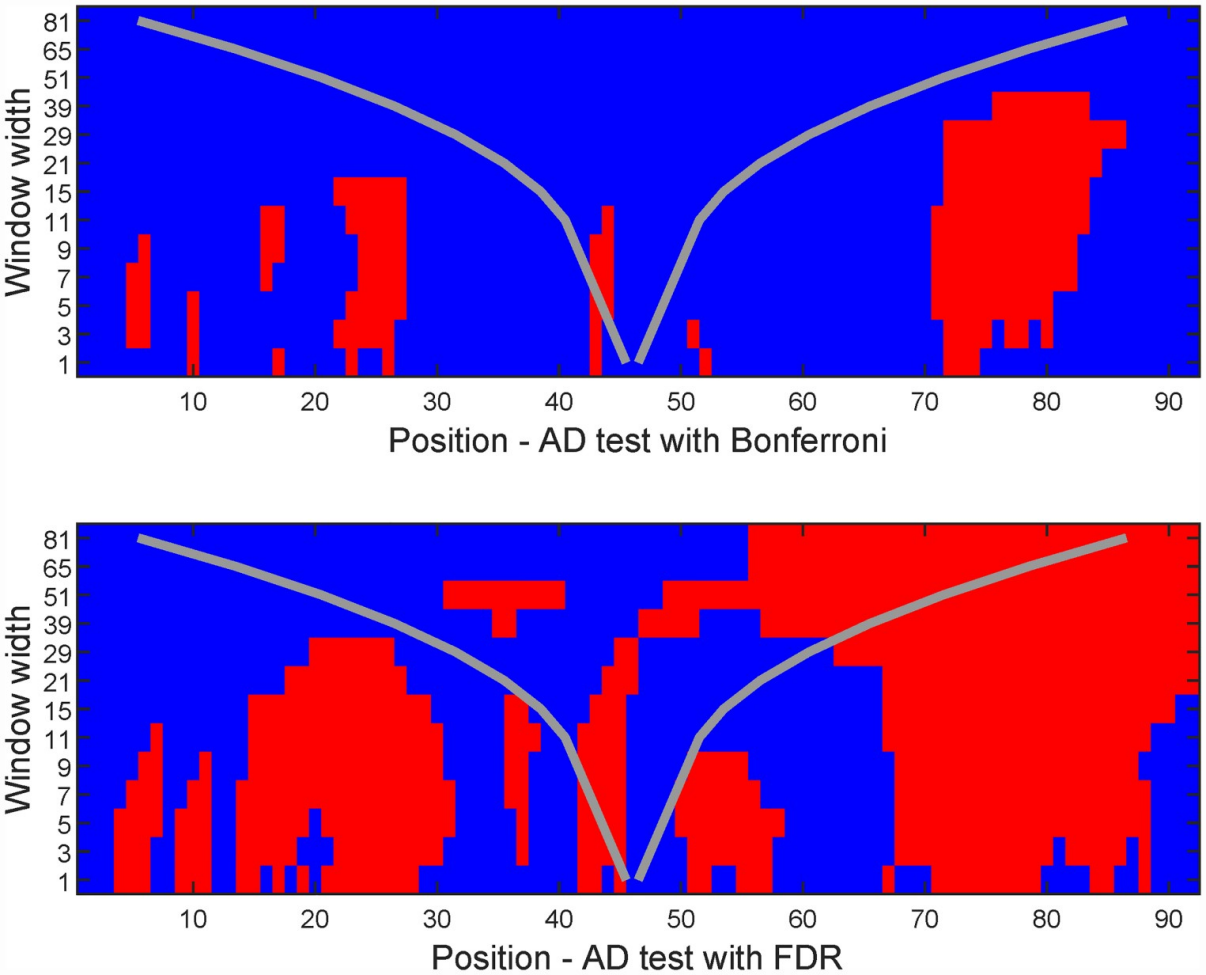
Significance maps for summer temperatures in Oslo. See Fig 3 for annotation details.

**Fig 9:**
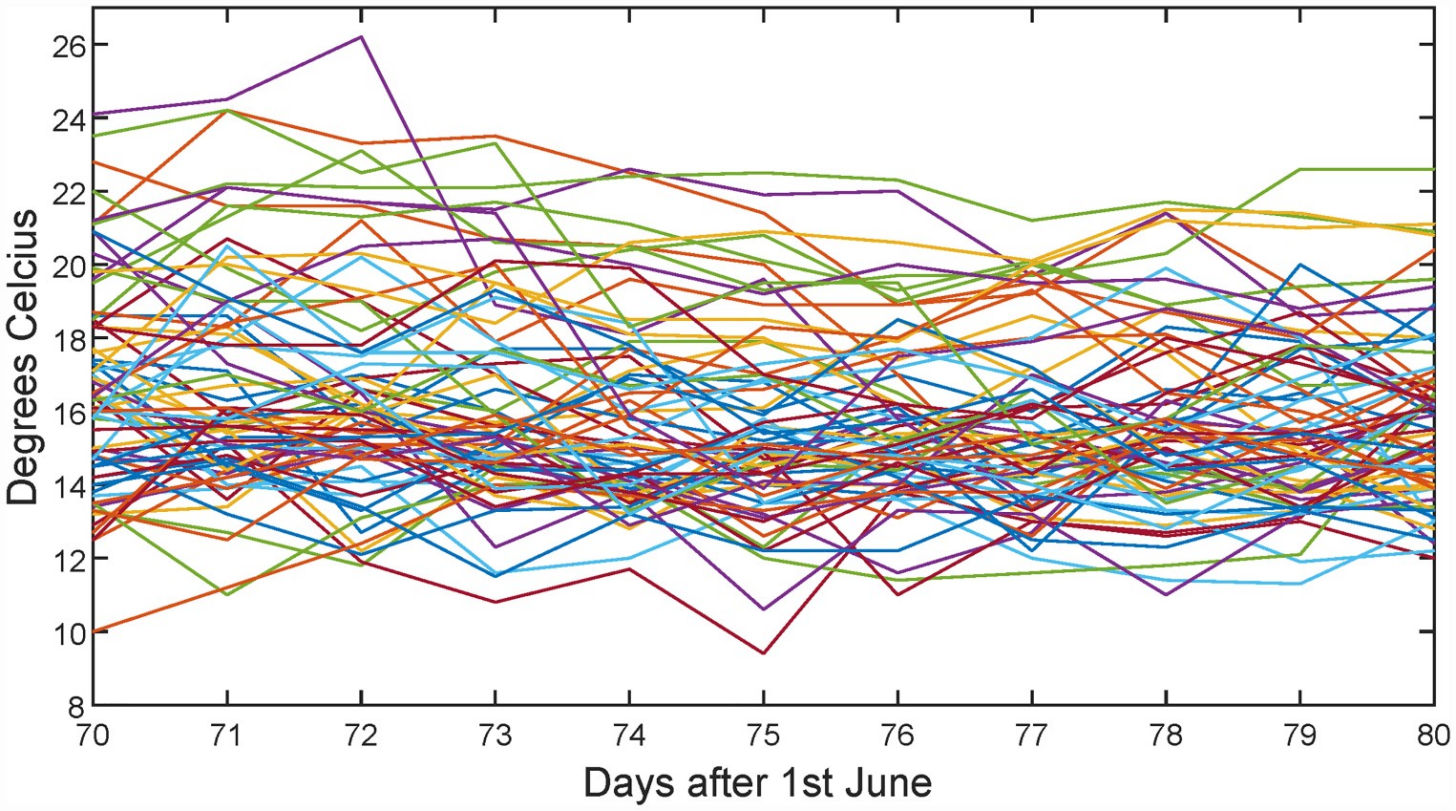
Mid-August temperatures in Oslo for the years 1937–2008.

Fig 8 gives the multinormality check results. Note that significant features are found both for the FDR and Bonferroni correction. To see what is going on, the period around time point 75 (i.e. in the middle of August) is shown in Fig 9. From this figure it seems that the mean temperature is around 15°C, but the temperature distribution around this time is skewed upwards. This means that Oslo at this time of the year experiences larger positive than negative deviations from the mean, which is not a surprising result if you have knowledge about the climate in that area.

### Comparison of temperature records

**Fig 10:**
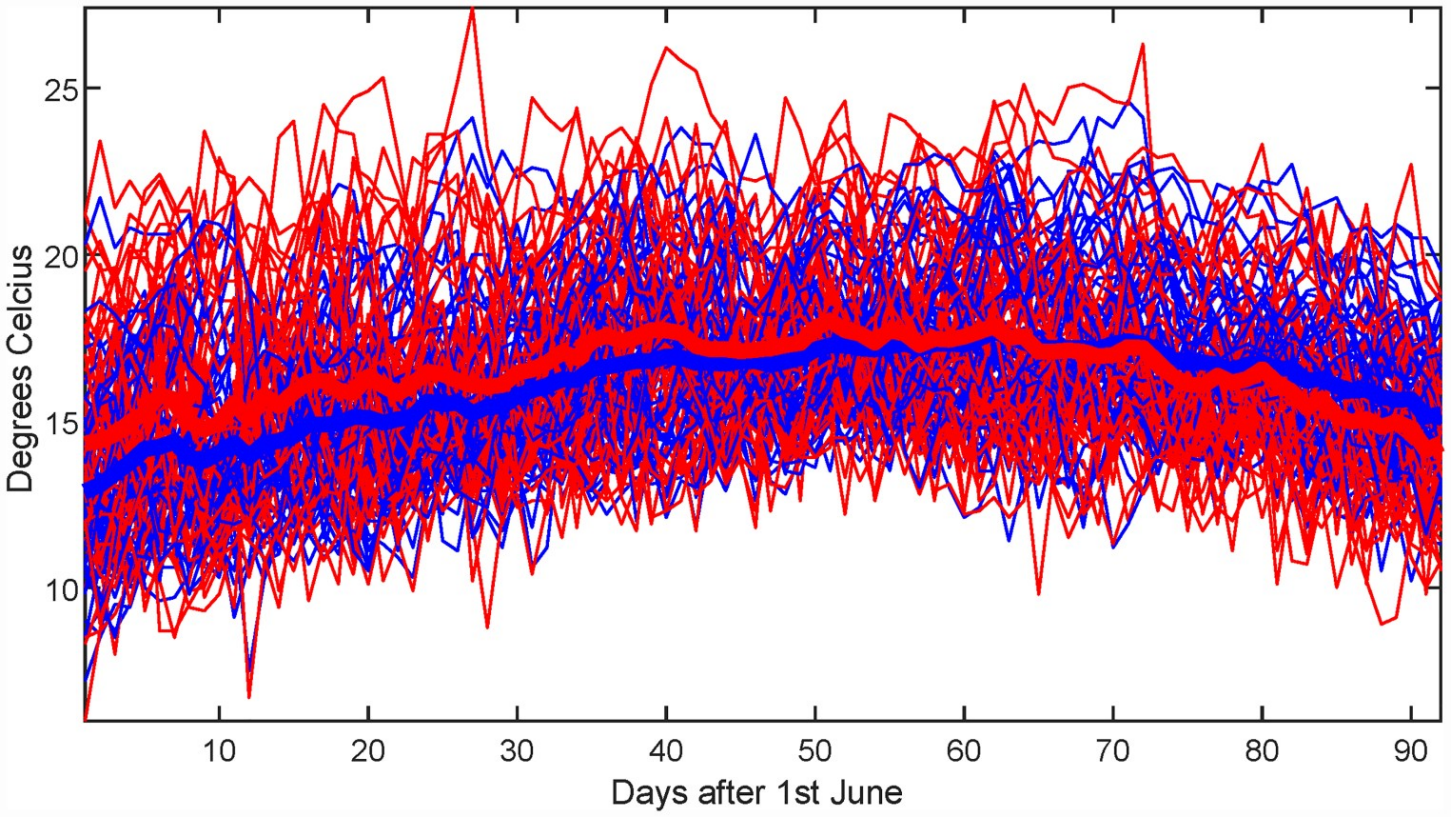
Temperature data from Ferder (blue) and Fornebu (red) and mean values marked by thick lines.

**Fig 11:**
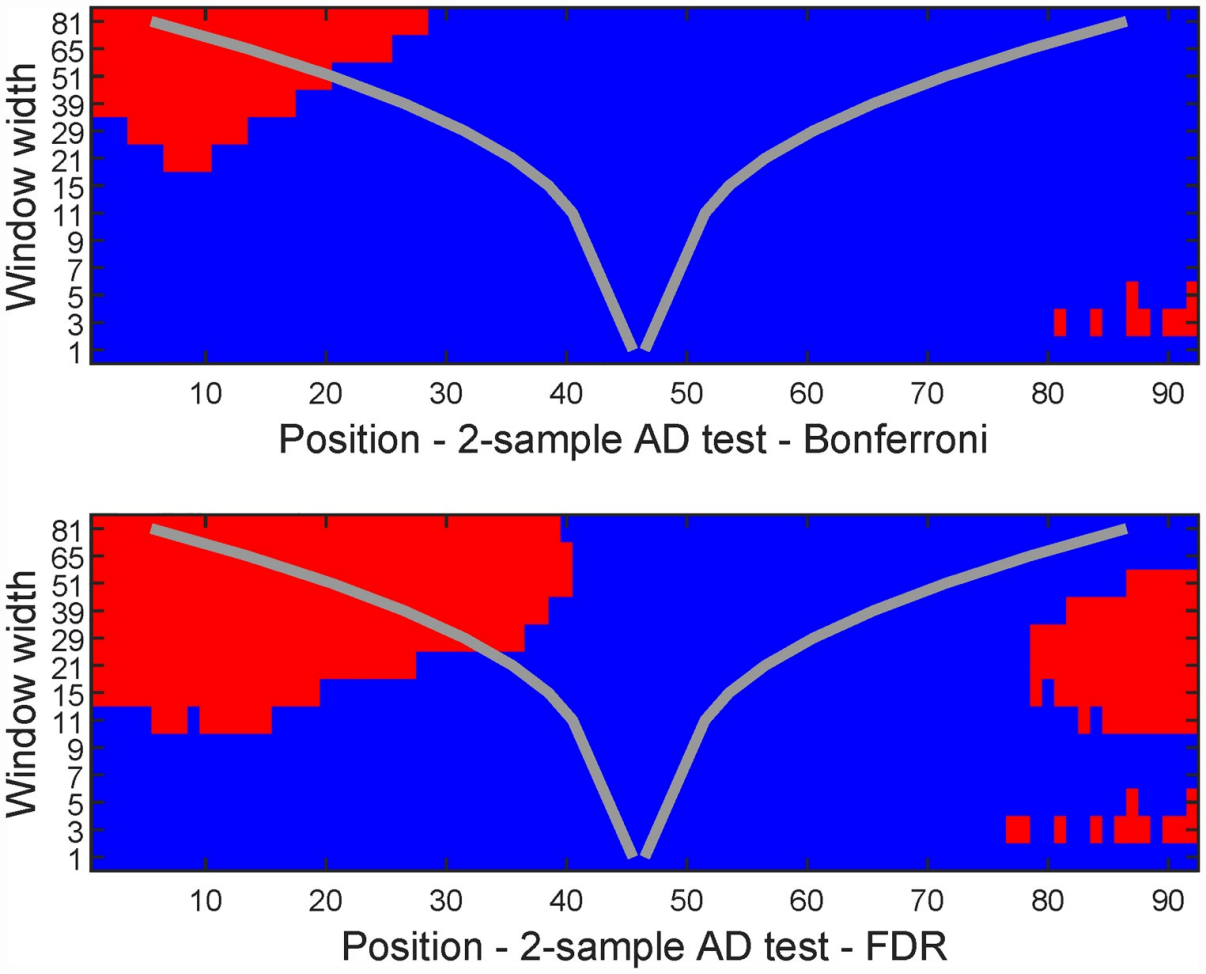
Significance maps from comparing the temperature data of Ferder and Fornebu with the scale-space method. See Fig 3 for annotation details.

Temperature data sets from two different meteorological stations in the Oslo area are compared. One is located at Ferder lighthouse at the start of the 100 km long Oslo fjord, while the other is located at Fornebu, which is at the very inner part of the Oslo fjord. The two data sets consist of more or less overlapping yearly records, with 64 and 45 complete years, respectively. Years with missing data in the months of interest have been removed. Fig 10 shows the two data sets, and Fig 11 shows the resulting significance maps. It is clear that the temperature distribution at the two stations differ early and late in the summer. From a closer inspection, it is clear that Fornebu is warmer in early summer, while the opposite effect takes place a few months later.

### Comparison to other methods

Just about all methods for testing for multinormality rely in some way on inverting the estimated covariance matrix. When the number of samples is less or equal to the number of dimensions (HDLSS setting), i.e. when *n* ≤ *p*, the estimated covariance matrix is non-invertible. The projection methods of [41] and the method based on Srivastava’s graphical method in [42] are applicable in this HDLSS setting, but no open implementations of the methods exist for power evaluation. The methods of Liang in [43, 44] are also applicable in the HDLSS setting and open implementations exist. The preferred method of Liang [43] first transforms the data matrix, and then projects it onto some lower-dimensional space of dimension *d* ≤ min(*n* − 2, *p*). The transformed data will under the null hypothesis be distributed as a *d*-dimensional standard multinormal vector, something which is checked using the skewness and kurtosis test of [45]. Asymptotic distributions are given, but in the setting of interest (*n* is not large compared to *p*), the use of the Liang test [43] relies on a permutation procedure for generating p-values.

It is not straightforward to compare the presented scale-space method to the Liang procedure since the presented scale-space method does not produce one single answer to the hypothesis testing problem. A simple example is analyzed to illustrate that the presented method outperforms the Liang test in some settings. Assume the same data set structure as in the example of the Introduction, except that the only non-normal part is the mixture of dimensions 6 to 12, the other dimensions are zero mean normally distributed. This setup results in the optimal resolution/position pair being (4, 9), i.e. summing over dimensions 6 to 12. When the non-zero mean value in this area is 2.35, the presented scale-space method has a detection ratio of 0.884/0.918 (Bonferroni/FDR) for the pair (4, 9) (based on 1000 Monte Carlo repetitions). The Liang test has for the same data sets a rejection ratio of 0.659. For the Liang test only the kurtosis test and only the optimal projection dimension (*d* = 1) are used. In a real setting, the optimal projection dimension would not be known and both the skewness and kurtosis test would be used, leading to a significantly lower power when the correction for multiple testing is done. In the same way, when the non-zero mean value is 2.05, the presented scale-space method has a rejection ratio of 0.569/0.628 for the pair (4, 9), while the Liang test has for the same data sets a rejection ratio of 0.480.

For the comparison of two or more data sets, there are several methods that handle the *n* ≤ *p* situation. Many of these methods use some kind of distance measure between the data vectors [46–49]. From these distances, the test statistics are generated, without estimating any covariance matrices. The test by Székely and Rizzo [49] is a *k*-sample extension of the two-sample test suggested by Baringhaus [50]. A similar two-sample test was suggested by Aslan [51]. The Aslan test performed very similar to, but not better than, the Székely-Rizzo/Baringhaus test in the two-sample test case of Table 2. Different projection methods that handle the *n* ≤ *p* situation also exist, e.g. Random Projection (RP) [52] and DiProPerm [53] (see paper for more methods). For the case of interest (*n* ≤ *p*), the tests all rely on permutation procedures to determine the p-value of the test statistic.

**Table 2:** Power of comparing a number of different data sets with a varying number of dimensions (“Dim”) for which there is an expected value difference *δ* in the tested data sets. For the Hall test, the *T* and *S* tests gave very similar results. Three nearest neighbors were used in the Nearest Neighbor test. The results of the Friedman-Rafsky test are for three trees, which consistently performed better than one and two trees in this setting. The scale-space results are for the Bonferroni/FDR correction, respectively. A 0.10 significance level is used and 2000 Monte Carlo samples are used.

	Dim: 1	Dim: 3	Dim: 5	Dim: 7
**Two-sample**	***δ* = 0.85**	***δ* = 0.75**	***δ* = 0.65**	***δ* = 0.55**
Scale-space	0.579/0.591	0.725/0.746	0.722/0.782	0.612/0.725
Friedman-Rafsky [46]	0.273	0.513	0.588	0.570
Hall-Tajvidi [47]	0.166	0.394	0.515	0.513
Nearest Neighbor [48]	0.256	0.487	0.543	0.531
Székely-Rizzo [49]	0.400	0.789	0.866	0.843
RP [52]	0.286	0.410	0.425	0.408
DiProPerm [53]	0.465	0.551	0.518	0.444
**Three-sample**	***δ* = 0.45**	***δ* = 0.35**	***δ* = 0.325**	***δ* = 0.30**
Scale-space	0.608/0.631	0.605/0.633	0.697/0.759	0.719/0.805
Székely-Rizzo	0.330	0.575	0.740	0.807
**Seven-sample**	***δ* = 0.15**	***δ* = 0.11**	***δ* = 0.10**	***δ* = 0.09**
Scale-space	0.731/0.745	0.611/0.633	0.695/0.740	0.672/0.757
Székely-Rizzo	0.295	0.468	0.622	0.675

The case of two data sets *X* and *Y* is first investigated. The expected value of *X* is zero for all dimensions, while *Y* has one region of a number of neighboring dimensions with a non-zero expected value. Both *X* and *Y* have the same covariance structure as the example of the Introduction. The number of dimensions of *Y* that have a non-zero mean value is varied, along with this non-zero value. The upper part of Table 2 shows the results. The result of the scale-space algorithm refers to the resolution/position pair with the highest rejection ratio.

Of the alternative tests, the method of Székely and Rizzo [49] consistently shows the greatest power in the tested settings. When the difference between *X* and *Y* is across many dimensions, the power of the Székely and Rizzo test is higher than the power of the scale-space approach. If there instead is only one dimension with a different distribution of *X* and *Y*, the power of the scale-space approach is greater than for the Székely test. This means that the Székely is a good alternative approach, but by using the scale-space approach one can determine where in the data set the difference is located.

For the case of *k* = 3, the presented scale-space method is only compared to the method of Székely and Rizzo (the Hall-Tajvidi, RP and DiProPerm tests cannot be extended to *k* > 2). The same covariance structure as for the two-sample case is used for the three data sets *X*, *Y* and *Z*. *X* is zero mean, while *Y* has for some neighboring dimensions a non-zero expected value of ***δ***, and *Z* has for the same dimensions a non-zero expected value of −***δ***. See the middle part of Table 2 for the results. The case of *k* = 7 is finally investigated in the lower part of Table 2. Here, the different data sets have the same structure as for the case of *k* = 3, but the different data sets *X*_*i*_, *i* = 1, 2, …, 7 have mean values equal to *i* ⋅ ***δ*** for the non-zero dimensions. From these results, the scale-space method seems to improve compared to the Székely-Rizzo method when the number of data sets increase, and the methods are giving comparable results in the tested settings.

### Feature selection

In a classification setting, the p-values of the different resolution/position pairs can be used to find useful scale-space features. The pairs with the smallest p-values should be good candidate features for classification algorithms. The p-values of neighboring pairs will be correlated (for all resolution values larger than 1). An ad hoc strategy to avoid the selection of neighboring pairs is used. That is, say that the most significant pair is at window width 7 (i.e. resolution number 4) and position 5. Then, all pairs for two resolutions down (resolution number 2 and 3) and two resolutions up (resolution number 5 and 6) that sum over the data of position 5, are excluded from being selected as a feature as a result of pair (4, 5) being selected as a feature. The next feature to be selected corresponds to the resolution/position pair, which has not been excluded in the steps before, with the lowest p-value of the pairs not already selected. This is repeated until a wanted number of features are found or there are no good features left to pick from, where a potential feature’s “goodness” is connected to its p-value.

The suggested feature selection algorithm is tested on a setting similar to the example of the Introduction. Here, instead of having one data set with two parts, there are two data sets *X* and *Y*. *X* is distributed as the 20 first samples of the motivational example, while *Y* is distributed as the 20 remaining samples, except that the expected value equals −0.65 for position index 6 to 12 and −1 for position index 20. For indices 26, …, 40, the expected value increases linearly from 0.05 to 0.75.

**Fig 12:**
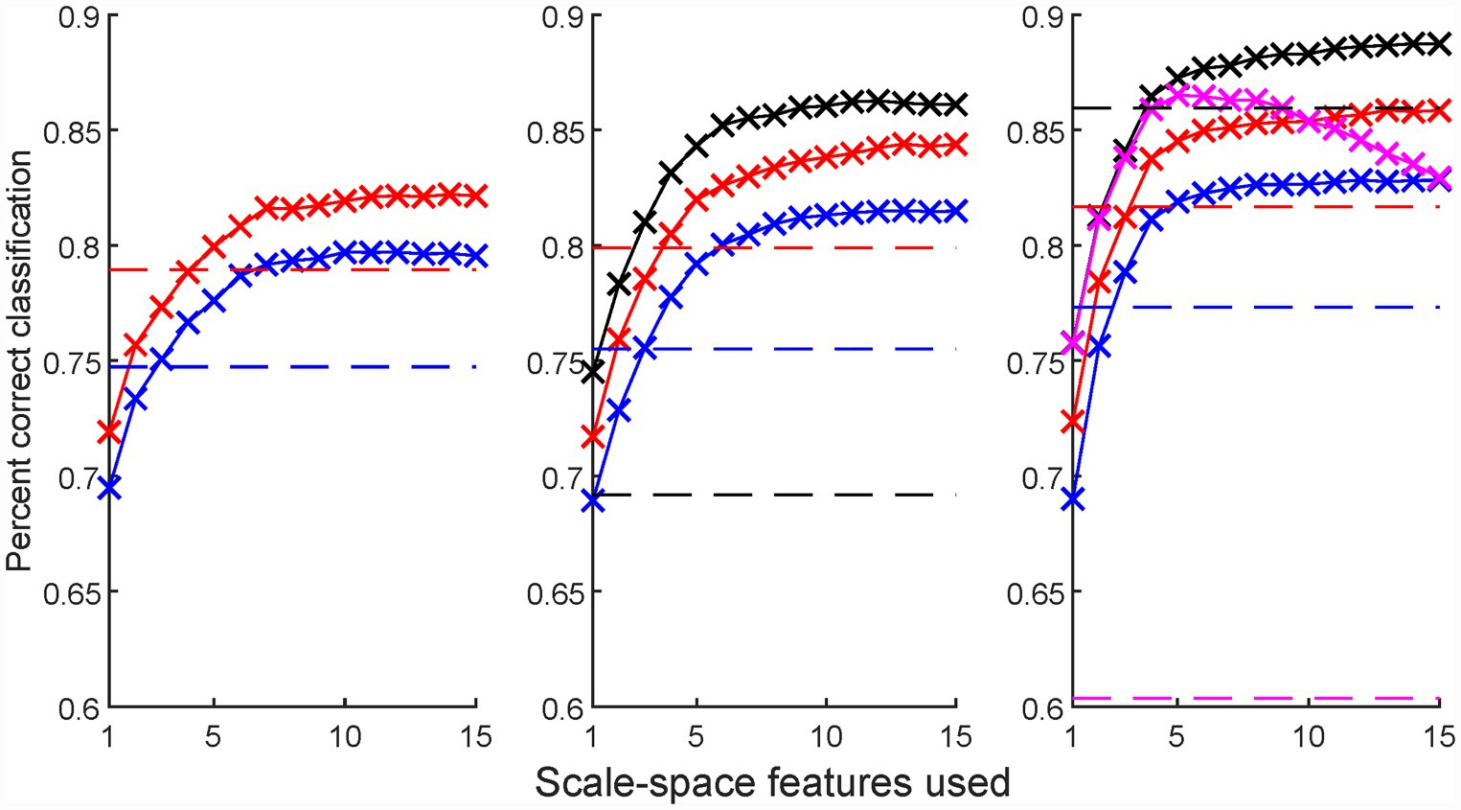
Classifcation results when using scale-space features (solid lines) and all dimensions (dashed lines). Classification methods are given as 1NN (blue), 3NN (red), LDA (black), QDA (magenta). The vertical axis shows the ratio of correct classifications based on 100000 simulations. Sample sizes from left to right are: 20, 30 and 60.

The suggested feature selection scheme is compared to using all dimensions as inputs to classification algorithms. This is meant as a proof of concept, not a thorough comparison to other methods. The tested sample sizes of both *X* and *Y* were 20, 30 and 60. For the classification, *k* Nearest Neighbor classification (with *k* = 1 and *k* = 3), Linear Discriminant Analysis (LDA) and Quadratic Discriminant Analysis (QDA) were used, when applicable [54]. For the scale-space feature selection, the number of features selected ranged from 1 to 15. One pair of *X* and *Y* data sets was used to find the training features. These features were then used to classify 500 *X* and 500 *Y* data sets. This was repeated 100 times, making up in total 100000 tests, and the ratio of correct classification was averaged across these 100000 tests, as shown in Fig 12. The splitting up was done to average out the fact that different features will be selected depending on the training data set. With three well-selected features, one can capture the main differences in the two data sets, but as the figure shows, one needs on average more than three features to have the maximum ratio of correct classification. The figure shows that using the suggested scale-space features is better than using the raw data in this example.

## Conclusions

The scale-space methodology is applied to the testing for multivariate normality and the *k*-sample problem. The summation across dimensions/positions reduces the multivariate problem to a large number of one-dimensional tests. A significance map, showing where and for which resolutions the null hypothesis is rejected, is generated by going through all combinations of the position and resolution parameters. The summation throws away all information of the dependency structure of the data. When there are more multivariate observations than dimensions, i.e. *n* > *p*, the discharging of covariance information will lower the power of the scale-space tests compared to tests that use this information gained through estimation of the covariance matrix. What is gained on the other hand, is the ability to check for multinormality and compare data sets in the High Dimension Low Sample Size setting, something which almost all other methods fail to handle.

The presented algorithms are tested on artificial data and real temperature data sets, showing how both the check for multinormality and how the comparison of data sets can be done through a scale-space approach.

Within the scale-space framework, to the authors’ best knowledge, there is no other algorithm to compare the presented work with, even though a large number of tests for assessing the multinormality of a given data set exist [26, 55–57]. To the knowledge of the authors, the only multivariate methods for testing multinormality that handle the case when *n* ≤ *p*, are the methods [41–44]. The preferred Liang method [43] is inferior to the presented method in some relevant aspects and cases.

In the case of comparing *k* data sets, there exist some methods that handle the case where at least one of the sample sizes are less than the number of dimensions. In general, these methods are based on some distance measure between the data vectors, and do not estimate the covariance matrix, or projection onto lower-dimensional spaces. The suggested scale-space method is compared to these methods. In the tested settings, the power of the method of Székely and Hall [49] is comparable to the power of the scale-space approach. The Székely test does not on the other hand provide any info about where the data sets differ, information that is essential for doing feature selection. Selection of relevant features based on the presented scale-space *k*-sample problem algorithm is demonstrated in the “Results” section.

## Supporting information

S1 File MATLAB-files for running the presented algorithms. (ZIP)

S2 File Data sets used in paper. (ZIP)
